# Evidence Suggests Prophylactic Antibiotics May Be Unnecessary in Anorectal Surgery—A Systematic Review and Meta‐Analysis

**DOI:** 10.1002/wjs.70255

**Published:** 2026-02-05

**Authors:** James Jin, Velia Men, Maggie Wang, Andrew Hill

**Affiliations:** ^1^ Department of Surgery South Auckland Clinical Campus Middlemore Hospital Auckland New Zealand; ^2^ Faculty of Medical and Health Sciences The University of Auckland Auckland New Zealand

**Keywords:** anorectal diseases, antibiotic prophylaxis, hemorrhoidectomy, surgical site infection

## Abstract

**Background:**

Amid increasing global concerns regarding antimicrobial resistance, the routine use of prophylactic antibiotics in anorectal surgery has been questioned. In practice, prescribing practices vary widely among surgeons, highlighting the need for stronger evidence‐based guidance. The aim of this study is to perform a systematic, critical assessment of the current literature to determine the role of prophylactic antibiotics in elective anorectal surgery.

**Methods:**

A comprehensive search of studies published between January 1980 and June 2025 was performed using PubMed, Embase, and Cochrane Library. The primary outcome was surgical site infection (SSI); secondary outcomes included systemic infection, wound dehiscence, abscess formation, bleeding, and recurrence. Study quality was assessed using the Cochrane RoB 2.0 tool for randomized controlled trials (RCTs) and the ROBINS‐I tool for observational studies. The certainty of evidence was evaluated using the GRADE approach.

**Results:**

Nine studies including 2317 participants were included, and five were eligible for meta‐analysis. Overall, prophylactic antibiotics were not associated with a significant reduction in postoperative infectious or wound‐related complications in RCTs (RR 0.76, 95% CI 0.43–1.33, and *p* = 0.66, moderate GRADE certainty of evidence) or observational studies (RR 0.60 (95% CI 0.01–48.4) and *p* = 0.53, very low certainty). All studies concluded that routine antibiotic prophylaxis may be unnecessary in anorectal surgery.

**Conclusion:**

Current evidence does not support the routine use of prophylactic antibiotics in uncomplicated anorectal procedures. However, the certainty of evidence is limited by small sample sizes, methodological heterogeneity, and limited number of available studies. Large‐scale randomized trials are required to strengthen this evidence base.

**Trial Registration:**

The review protocol was registered in the PROSPERO database CRD420251159850

## Graphical abstract

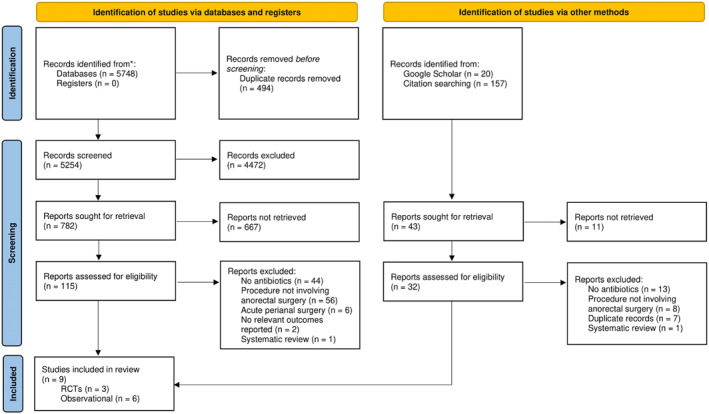

## 1 Introduction

Antibiotics are essential in modern surgery and are widely used to prevent surgical site infections (SSIs). However, a key challenge today is balancing effective prophylaxis with the risks of antibiotic overuse, which contributes to the growing issue of antimicrobial resistance [1]. In surgery, antibiotic resistance leads to prolonged hospitalization, extended treatment, higher mortality, and increased reoperation rates [2]. Therefore, re‐evaluating the need for antibiotics in specific surgical settings is critical to ensure safe, appropriate, and evidence‐based care.

Although prophylactic antibiotics are well established in many surgeries, their routine use in lower risk procedures, such as elective anorectal surgery, remains controversial. Anorectal procedures are associated with lower infection rates, but complications including SSIs can occur and may lead to serious outcomes, including wound dehiscence, abscess formation, and sepsis. At the same time, antibiotics carry inherent risks. Up to 20% of patients may experience antibiotic‐associated adverse effects, including gastrointestinal symptoms, allergic reactions, and *Clostridioides difficile* infection although this rate can vary depending on the antibiotic used and the detection method [3].

Currently, there is no clear consensus regarding the use of prophylactic antibiotics in anorectal surgery. Existing clinical guidelines are conflicting; some guidelines recommend against routine antibiotic use in uncomplicated cases, but these are often based on inconclusive evidence and are not universally accepted [4, 5]. Consequently, prescribing practices vary widely, with reported antibiotic use ranging from 40% to 56% across different institutions and surgeons [6, 7].

This study aimed to perform a systematic review and synthesis of current evidence on the role of prophylactic antibiotic use in elective anorectal surgery. The goal was to clarify the benefits and necessity of antibiotic prophylaxis in this context to help inform evidence‐based clinical practice.

## 2 Methods

### 2.1 Information Sources

A systematic review and meta‐analysis was conducted per the Preferred Reporting Items for Systematic Reviews and Meta‐Analyses guidelines (Supporting Information S1: Table S1) using MEDLINE, Embase, and Cochrane Library.

### 2.2 Search Strategy

A comprehensive search strategy combining Medical Subject Heading (MeSH) terms and keywords was used to identify relevant studies, including: “antibiotic prophylaxis,” “anus diseases,” “rectal diseases,” “anal canal,” “hemorrhoidectomy/hemorrhoidectomy,” “hemorrhoids,” “rectum,” “anal fistula,” “rectal fissure,” “anal fissure,” “fissure in ano,” “sphincterotomy,” “fistulectomy,” and “fistulotomy.” The full search strategy is shown in Supporting Information S1—Figure S1.

### 2.3 Eligibility Criteria

We included studies published in English between January 1980 and June 2025 that examined prophylactic antibiotic use in adults (≥ 18 years) undergoing elective anorectal surgery. Eligible procedures involved the distal rectum, anus, or perianal skin, including but not limited to hemorrhoidectomy, fistulectomy, fistulotomy, fissurectomy, and sphincterotomy. Studies needed to report antibiotic use (pre‐, intra‐, or postoperative) and postoperative infection–related complications such as SSIs, wound dehiscence, abscess formation, secondary (delayed) bleeding, or systemic infection. Both randomized clinical trials (RCTs) and observational studies were included.

Studies were excluded if they involved procedures outside the anorectal region, such as intraperitoneal colorectal or abdominal surgeries. Acute perianal procedures, such as incision and drainage of perianal abscesses, and pilonidal surgery, were also excluded as they involve already infected tissue with a higher baseline infection risk, different tissue characteristics, and therapeutic rather than prophylactic antibiotic use. Minimally invasive or nonsurgical procedures, such as hemorrhoidal artery ligation, botulinum toxin injection, laser surgery, and endoscopic banding were excluded because they do not involve significant tissue disruption and generally carry a very low infection risk. Studies in which antibiotics were administered for purposes other than infection prophylaxis, such as metronidazole for postoperative pain, were also excluded. Furthermore, studies were excluded if they did not report postoperative outcomes or if they were single case reports, case series, conference abstracts, or narrative reviews. Studies focusing on highly specific populations, such as obstetric anorectal procedures, were excluded to maintain the generalizability of the findings.

### 2.4 Selection and Data Collection

Two reviewers independently screened the titles and abstracts. The reference lists of studies and systematic reviews were also searched for additional records. Data extraction was conducted by two independent reviewers using a predefined form, including study design, setting, sample size, study period, follow‐up duration, surgical procedure, antibiotic regimen (timing, agent, and duration), and postoperative outcomes. Noninfective outcomes, such as postoperative pain and urinary retention, were not included, as they are unlikely to be directly influenced by antibiotic prophylaxis. Early postoperative bleeding occurring within 24 h of surgery was excluded as it is primarily attributable to the surgical procedure and unlikely related to infection.

### 2.5 Risk of Bias Assessment

We assessed the risk of bias using the ROBINS‐I tool for nonrandomized studies and the Cochrane Risk of Bias (RoB) 2.0 tool for RCTs [8, 9]. A sensitivity analysis was conducted to test the robustness of the study effects to the exclusion of RCTs or non‐RCTs. Publication bias was assessed using funnel plots generated using RStudio.

### 2.6 Statistical Analysis

Meta‐analyses were performed using the *meta* package in RStudio. We conducted separate meta‐analyses of RCTs and non‐RCTs using subgroup analysis and meta‐regression to assess potential interactions between study design and summary effects. Pooled risk ratios (RR) with 95% confidence intervals (CI) were calculated using a random effects model with inverse variance weighting. Continuity correction was applied to studies with zero events in both arms. Pooled event proportions for the intervention and control groups were calculated using logit transformation under a random effects model. Statistical heterogeneity was assessed using the I^2^ statistic.

### 2.7 Certainty Assessment

Evidence certainty was assessed using the GRADE approach, and absolute effects were calculated using the GRADEpro tool [10].

## 3 Results

**FIGURE 1:**
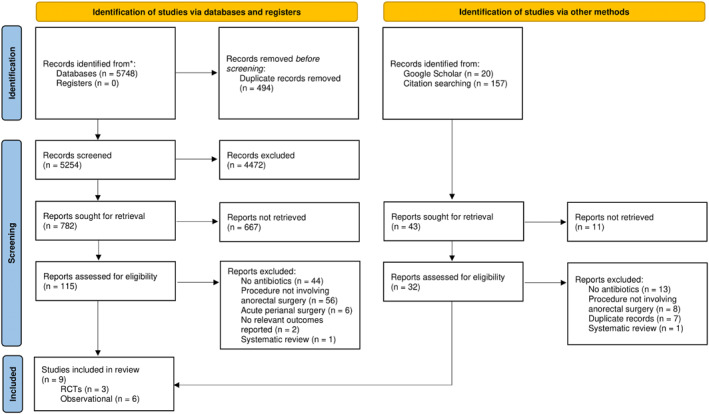
PRISMA flow diagram. *Source:* Page MJ, McKenzie JE, Bossuyt PM, Boutron I, Hoffmann TC, Mulrow CD, et al. The PRISMA 2020 statement: an updated guideline for reporting systematic reviews. *BMJ*. 2021; 372:n71. 10.1136/bmj.n71.

From the 6033 search results, nine studies were included in this review, including three RCTs and six observational studies (Figure 1). These studies included 2317 participants, with a mean age of 43.8 years, and 39.6% were female. Sample sizes ranged from 75 to 852 participants (mean 257). Study characteristics are summarized in Table 1.

**TABLE 1:** Characteristics of the included studies.

Author	Study design	Procedure(s)	Sample size (*n*)	Mean age (yrs)	Female (%)	Antibiotic regimen	Intervention (*n*)	Control (*n*)	Outcome(s)	Follow‐up
Asif et al.	Prospective descriptive	Hemorrhoidectomy, fistulectomy, and lateral sphincterotomy	182	48.0	31.3	No antibiotics	0	182	SSI	2 months
Hasan et al.	Prospective longitudinal	Fistulotomy	75	40.0	8.0	Varied regimens, post‐op	75	0	SSI, sepsis, abscess, and recurrence	6 and 12 months
Hosseini et al.	RCT	Fissurectomy and lateral sphincterotomy	311	37.0	55.9	Metronidazole PO 1 week post‐op	156	155	SSI, wound dehiscence, and bleeding	2 weeks
Khan et al.	RCT	Hemorrhoidectomy	96	40.5	38.3	Metronidazole IV + ceftriaxone single dose pre‐op	47	49	SSI and sepsis	4 weeks
Liu et al.	RCT	Hemorrhoidectomy	150	44.1	56.0	Cefoxitin IV pre‐ and 6h post‐op	100	50	Sepsis, abscess, recurrence, and bleeding	Until healing
Memon et al.	Descriptive	Hemorrhoidectomy	200	41.0	40.0	No antibiotics	0	200	SSI and sepsis, bleeding	6 weeks
Nelson et al.	Retrospective cohort	Hemorrhoidectomy	852	50.0	50.1	Varied regimens, pre‐op	352	500	SSI and sepsis	≥ 12 weeks
Patel et al.	Retrospective cohort	Biopsy of anal lesion	275	41.6	12.0	Varied single‐dose IV pre‐op	38	237	SSI	≥ 1 month
Wesarachawit et al.	Retrospective matched pair	Hemorrhoidectomy	176	51.9	64.8	Metronidazole PO 7‐days post‐op	88	88	Wound dehiscence, bleeding	4 weeks

Abbreviations: RCT: randomized controlled trial; SSI: surgical site infection.

Procedures included hemorrhoidectomy (*n* = 6), lateral sphincterotomy (*n* = 2), and individual studies on fistulectomy, fistulotomy, fissurectomy, and biopsy of anal dysplasia. Reported outcomes included SSI (*n* = 7), systemic infection (*n* = 5), wound dehiscence (*n* = 2), abscess formation (*n* = 2), bleeding (*n* = 4), and recurrence (*n* = 2).

Antibiotic regimens varied widely, ranging from single preoperative doses to extended postoperative courses. Most studies used broad‐spectrum antibiotics targeting both aerobic and anaerobic organisms, typically combining metronidazole with a cephalosporin. Four studies used postoperative antibiotics, three used preoperative, and two included only patients who did not receive antibiotics. All studies used systemic intravenous or oral antibiotics.

Among 2317 participants, 130 postoperative infection or wound‐related complications were reported (Table 2). SSIs were the most common, reported in 4.13% (77/1866) of cases. Wound dehiscence, recurrence, abscess formation, and bleeding occurred at rates of 3.29%, 1.86%, 1.78%, and 2.99%, respectively. Sepsis in routine anorectal surgery was rare, affecting only 0.34% (4/1173) of patients.

**TABLE 2:** Postoperative infection‐related complications after elective anorectaly surgery.

Event	Author	No. of events	Sample size	Rate (%)
Surgical site infection	Asif et al.	0	182	0.00
	Hasan et al.	0	75	0.00
	Hosseini et al.	61	311	19.6
	Khan et al.	0	96	0.00
	Liu et al.	4	150	2.67
	Memon et al.	0	200	0.00
	Nelson et al.	12	852	1.41
	Patel et al.	2	237	0.80
	Total	79	2103	4.13
Systemic infection	Hasan et al.	0	75	0.00
	Khan et al.	0	96	0.00
	Liu et al.	4	150	2.67
	Nelson et al.	0	852	0.00
	Total	4	1173	0.34
Wound dehiscence	Hosseini et al.	12	311	3.86
	Wesarachawit et al.	4	176	2.27
	Total	16	487	3.29
Recurrence	Hasan et al.	0	75	0.00
	Liu et al.	4	140	2.86
	Total	4	215	1.86
Abscess formation	Hasan et al.	4	75	5.33
	Liu et al.	0	150	0.00
	Total	4	225	1.78
Bleeding	Hosseini et al.	18	311	5.79
	Liu et al.	5	150	3.33
	Memon et al.	0	200	0.00
	Wesarachawit et al.	2	176	1.14
	Total	25	837	2.99
	Total events	130	2317	5.61

### 3.1 Meta‐Analysis

**FIGURE 2:**
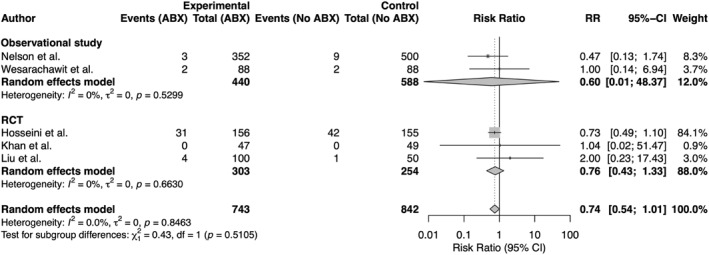
Forest plot of postoperative complications comparing antibiotic use versus no antibiotics. Studies are stratified by designs: observational studies (top) and randomized controlled trials (RCTs) (bottom). Antibiotic prophylaxis was associated with a nonsignificant reduction in infection risk overall.

Five studies qualified for the meta‐analysis, including three RCTs (*n* = 557) and two non‐RCTs (*n* = 1028). Overall, antibiotic use showed a nonsignificant reduction in postoperative complications compared with no antibiotics (RR 0.74, 95% CI 0.54–1.01, *p* = 0.85, and I^2^ = 0%–79.2%). Separate analysis by study design showed pooled RRs of 0.76 (95% CI 0.43–1.33 and *p* = 0.66) for RCTs and 0.60 (95% CI 0.01–48.4 and *p* = 0.53) for non‐RCTs, with no significant difference between study designs (*p* = 0.51). The corresponding forest plot is shown in Figure 2.

Qualitative synthesis supported our findings. All studies concluded no significant benefit of prophylactic antibiotics in elective anorectal surgery. Detailed results for individual outcomes are described below, and a summary of findings is provided in Table 3. Forest plots for individual outcomes are available in the Supporting Information S1. Overall, the certainty of evidence ranged from moderate to low, primarily due to low precision in effect sizes and risk of bias in nonrandomized studies.

**TABLE 3:** Summary of findings and GRADE evidence table.

Certainty assessment							No of patients		Effect		Certainty	Importance
No of studies	Study design	Risk of bias	Inconsistency	Indirectness	Imprecision	Other considerations	Prophylactic antibiotics	No antibiotics	Relative (95% CI)	Absolute (95% CI)		
Surgical site infection												
3	Randomized trials	Not serious	Not serious	Not serious	Serious^a^	None	30/303 (9.9%)	35/254 (13.8%)	RR 0.81 (0.55–1.20)	26 fewer per 1000 (from 62 fewer to 28 more)	⨁⨁⨁◯ moderate	Critical
1	Nonrandomized studies	Not serious	Not serious	Not serious	Serious^a^	None	3/352 (0.9%)	9/500 (1.8%)	RR 0.47 (0.13–1.74)	10 fewer per 1000 (from 16 fewer to 13 more)	⨁⨁◯◯ low	Critical
Systemic infection												
2	Randomized trials	Not serious	Not serious	Not serious	Very serious^d^	None	3/147 (2.0%)	1/99 (1.0%)	RR 1.37 (0.19–10.10)	4 more per 1000 (from 8 fewer to 92 more)	⨁⨁◯◯ low	Critical
Wound dehiscence												
1	Randomized trials	Not serious	Not serious	Not serious	Serious^a^	None	4/156 (2.6%)	8/155 (5.2%)	RR 0.50 (0.15–1.61)	26 fewer per 1000 (44 fewer to 31 more)	⨁⨁⨁◯ moderate	Important
1	Nonrandomized studies	serious^e^	Not serious	Not serious	Serious^f^	None	2/88 (2.3%)	2/88 (2.3%)	RR 1.00 (0.14–6.94)	0 fewer per 1000 (from 20 fewer to 135 more)	⨁◯◯◯ very low	Important
Bleeding												
2	Randomized trials	Not serious	Not serious	Not serious	Very serious^d^	None	14/256 (5.5%)	9/205 (4.4%)	RR 1.33 (0.00 to 8)		⨁◯◯◯ very low	Important
1	Non‐randomized studies	Not serious	Not serious	Not serious	serious^d^	None	1/88 (1.1%)	1/88 (1.1%)	RR 1.00		⨁◯◯◯ very low	Important

*Note:* Explanations (a) The confidence intervals around the effect estimates are wide and include both benefit and harm; the total number of events are smaller. (b) Results in the RCTs conflicted with substantial variability in pooled effects; this is reflected in the high statistical heterogeneity (I^2^ = 92.8%) and very wide confidence intervals. (c) Antibiotic regimens were nonstandardized and often based on surgeons' discretion, leading to potential confounding. (d) Wide confidence interval resulting from very few events in both groups and small sample sizes. (e) Risk of selection bias and confounding due to retrospective study design; exposure to antibiotics was not randomized and influenced by clinical judgment. (f) The total number of events and sample size was very low, and the confidence interval was wide, ranging from possible large reduction to a large increase in risk.

Abbreviations: CI: confidence interval; RR: risk ratio.

### 3.2 Surgical Site Infection

Three RCTs reported SSI rates after Milligan–Morgan hemorrhoidectomy (*n* = 2) and sphincterotomy with fissurectomy (*n* = 1) [11, 12, 13]. Antibiotics were associated with a modest but nonsignificant reduction in SSI rates (RR 0.81, 95% CI 0.55–1.20, and *p* = 0.15). Certainty of evidence was moderate and downgraded for imprecision due to small sample sizes and low event rates. None of the authors supported routine prophylactic antibiotic use for preventing SSI.

Observational evidence came from a large retrospective cohort study of 852 patients who underwent closed hemorrhoidectomy (Nelson et al.) [6]. Similar to the RCTs, the study found no significant benefit for SSI reduction with antibiotics (RR 0.47, 95% CI 0.13–1.74, and *p* = 0.17). However, due to its retrospective design and imprecision from rare events, the certainty of this evidence was low.

### 3.3 Systemic Infection

Data from two small RCTs showed no significant effects of antibiotics on systemic infection after Milligan–Morgan hemorrhoidectomy (RR 1.37 and 95% CI 0.19–10.10) [13, 14]. However, the certainty of this evidence was rated low due to imprecision from small sample sizes and very few events.

### 3.4 Wound Dehiscence

Two studies investigated the effect of postoperative metronidazole on the prevention of wound dehiscence. Both authors concluded that antibiotics do not significantly reduce the risk of wound dehiscence. Hosseini et al.’s RCT (*n* = 311) reported a nonsignificant reduction in wound dehiscence after internal sphincterotomy and fissurectomy (RR 0.73, 95% CI 0.49–1.10, and *p* = 0.50) [15]. Despite the low risk of bias, the certainty of evidence was rated low due to imprecision and reliance on a single trial.

Similarly, Wesarachawit et al. conducted a retrospective matched‐pair study of patients who underwent closed hemorrhoidectomy using the same antibiotic regimen [15]. Wound dehiscence rates were identical in each group (2.25%, RR 1.00, and 95% CI 0.14–4.94). However, the certainty of evidence was considered very low due to the study's moderate risk of bias and the small number of events.

### 3.5 Bleeding

Secondary postoperative bleeding was reported in four well‐designed studies, including two RCTs and two observational studies. None of the individual studies reported a difference in bleeding rates between the antibiotic and control groups. Pooled analysis of the two RCTs showed no significant risk difference (RR 1.33 and 95% CI 0.00–4818), although this estimate was highly imprecise due to the very small number of bleeding events and limited sample sizes.

### 3.6 Qualitative Analysis

Four studies were not eligible for meta‐analysis but were included in a qualitative synthesis. Asif et al. and Memon et al. observed no SSI in patients who underwent anorectal procedures without antibiotics, suggesting that routine prophylaxis may be unnecessary [14, 16]. In contrast, Hasan's study involved only patients who received postoperative antibiotics and reported 11.4% recurrence after fistulotomy and no cases of SSI or sepsis. The study concluded that antibiotics should be empirically given as they significantly reduce postoperative adverse events [17]. However, the lack of comparison groups in all three studies limited the strength of their conclusions due to the inability to draw causal inferences.

Patel et al. examined HIV‐positive patients who had undergone surgical biopsy of anorectal dysplasia. The study was excluded from the meta‐analysis due to limited comparability with other studies [18]. SSIs occurred in 0.8% (2/237) of procedures without antibiotics and 0% (0/38) with antibiotics, with no significant difference between the groups (*p* = 0.563). The authors concluded that antibiotics are not routinely indicated in HIV‐positive patients undergoing anorectal surgery with CD4 counts above 50. However, the study's narrow population and limited procedural scope limit its generalizability to more invasive surgeries and the broader surgical population.

### 3.7 Risk of Bias

**FIGURE 3:**
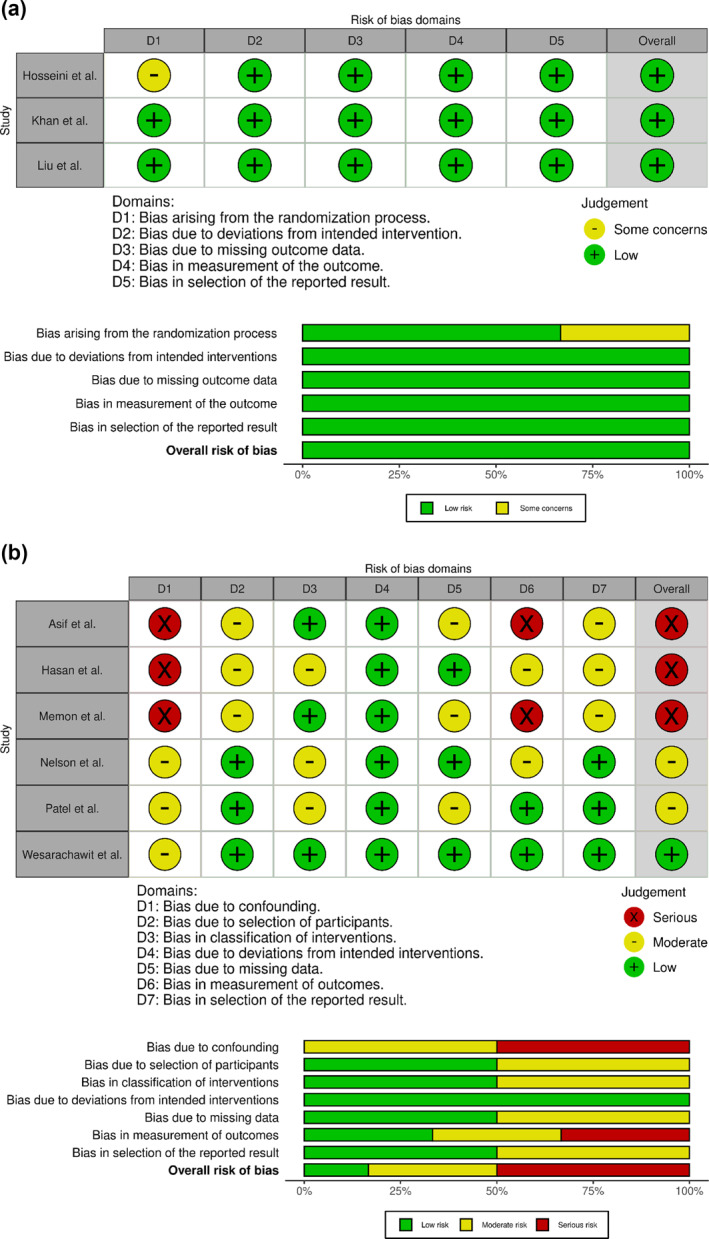
(a) Risk of bias in randomized controlled trials (RCTs) assessed using Cochrane Risk of Bias (RoB) 2.0. (b) Risk of bias in observational studies assessed using Cochrane ROBINS‐I.

All three RCTs were rated as having an overall low risk of bias (Figure 3a). The studies generally had robust randomization methods, low attrition, standardized antibiotic regimens, and objective outcome assessments using predefined clinical criteria. One study (Hosseini et al.) had some concerns as the randomization and allocation concealment methods were not clearly described.

Among the nonrandomized studies, three were rated as having a serious risk of bias, two as moderate, and one as low (Figure 3b). Key concerns included confounding, varied antibiotic regimens, and inconsistent outcome assessments. In all retrospective studies, antibiotics were not standardized and were often determined at the surgeon's discretion, increasing the risk of confounding by indication. Detection bias was also a concern in studies with subjective, unblinded outcome assessments, sometimes by the treating surgeon, with inconsistent definitions of wound infection.

Funnel plots demonstrated a roughly symmetrical distribution for RCTs (Supporting Information S1: Figure S4) and observational studies (Supporting Information S1: Figure S5), suggesting a low risk of publication bias. However, this interpretation is limited by the small number of included studies.

## 4 Discussion

This review provides a comprehensive analysis of the current evidence from nine studies on prophylactic antibiotic use in elective anorectal surgery. Meta‐analysis showed no statistically significant reduction in SSIs or other postoperative infectious or wound complications with antibiotic prophylaxis. Similarly, qualitative analysis provided limited support for routine antibiotic prophylaxis in uncomplicated procedures. These findings suggest that prophylactic antibiotics may be unnecessary in low‐risk anorectal procedures and could potentially cause more harm than benefits.

The rationale for antibiotic prophylaxis in anorectal surgery stems from the proximity of the region to dense microbial flora, creating a theoretical infection risk. However, the lack of significant benefit observed in this review is also biologically plausible. It is hypothesized that the perianal region's robust local immune defenses and high vascularity confer greater resistance to bacterial contamination than other body sites [19, 20]. Furthermore, anorectal procedures are generally short, less invasive, and relatively clean, resulting in a low inherent SSI rate, even in the absence of antibiotics. In this review, the overall SSI rate was 4.13%, which was lower than the rates reported for abdominal surgery (10%) and colorectal surgery (5%–20%) [21, 22, 23]. Given this low baseline risk, the absolute benefit of prophylactic antibiotics appears to be marginal, particularly when weighed against the potential adverse effects.

SSI risk varies with wound classification, ranging from 2.4% to 7.7% for clean‐contaminated wounds to 6.4%–15.2% for contaminated procedures [24]. Consequently, procedures such as incision and drainage of perianal abscesses carry a higher theoretical risk of infection than cleaner procedures like hemorrhoidectomy. Although some evidence suggests that the relative risk reduction of SSI from antibiotic prophylaxis may be the same across clean and higher risk procedures, data on the use of prophylactic antibiotics in perianal incision and drainage remain inconsistent [25]. Given the higher complication rates and theoretical contamination risk, future research should focus on these procedures in which prophylactic antibiotics may lead to greater clinical benefit.

Patient‐related factors, particularly diabetes status and glycemic control, are important considerations in postoperative infection risk and prophylactic antibiotic use. Glycemic control is a well‐established determinant of postoperative infection, as hyperglycemia impairs immune function and wound healing. Strict perioperative glycemic control is therefore a key preventive strategy in patients with poorly controlled diabetes and may be more significant in reducing infection risk than antibiotic prophylaxis alone. In this review, few studies reported diabetes prevalence, glycemic control, or stratified outcomes by diabetes status. Consequently, whether the benefit of prophylactic antibiotics differs in this higher risk subgroup remains unclear, and further targeted research is needed.

Our findings differ from the broader surgical literature, where prophylactic antibiotic use is well‐established. For example, a 2013 scoping review of 83 systematic reviews by Brocard et al. found strong evidence supporting preoperative antibiotic use across major surgical categories [26]. However, they also identified significant research gaps for specific procedures. Currently, there are no consensus guidelines or systematic reviews on antibiotic prophylaxis in anorectal surgery. International guidelines, such as those from the World Health Organization (WHO), recommend preoperative antibiotics mainly for major surgeries and do not address anorectal surgery specifically [27]. Extrapolating general surgical principles is limited by key contextual differences such as anatomical site, contamination risk, surgical technique, and patient populations, highlighting the need for procedural‐specific recommendations.

In practice, antibiotic use in surgery is often inconsistent and not always aligned with evidence‐based guidelines [28, 29]. Current guidelines recommend initiating antibiotics within 60–120 min before incision, with no routine continuation after surgery. Despite this, many patients still receive postoperative antibiotics—including four of the nine studies in this review and 60% of patients reported in the broader literature [30]. Observational data from this study also showed considerable variability in whether prophylactic antibiotics were given, as well as in timing, dose, and agent, highlighting ongoing uncertainty and inconsistency in clinical practice.

This review represents the most comprehensive synthesis to date of antibiotic use in anorectal procedures, including both RCTs and non‐RCTs across a range of outcomes. Strengths include methodological rigor, a systematic literature search, and the use of validated risk of bias assessment tools. Nonetheless, the review was limited by the small number and sample size of RCTs, heterogeneity among studies, and imprecision, which together reduced the overall certainty of findings.

Significant heterogeneity was observed across studies due to differences in antibiotic regimens, procedure types, adjuvant infection prevention measures, and patient populations. The quality of observational data was limited by methodological concerns, particularly measurement bias and confounding inherent to nonrandomized studies. Additionally, the clinical impact of postoperative complications, such as whether patients actually required treatment or reoperation, was seldom reported. Future studies should focus on well‐designed, adequately powered studies using standardized antibiotic protocols to improve the certainty of evidence.

The review has important implications for clinical practice. It suggests that prophylactic antibiotics may not be routinely necessary for elective anorectal procedures, challenging the common practice of universal prescribing. In reality, clinical decisions are rarely guided by evidence alone; patient factors, institutional policies, system constraints, and medicolegal concerns all have a significant influence on real‐world practice. Nonetheless, the evidence supports a selective, patient‐centered approach, reserving antibiotics for higher risk procedures or patients with significant risk factors. For certain groups, such as those with poorly controlled diabetes, immunocompromise, valvular heart disease, or prosthetic material in situ, antibiotics should still be considered on an individual basis. However, for the majority of patients undergoing routine, uncomplicated procedures, antibiotics can likely be safely omitted.

## 5 Conclusion

In conclusion, this review found no consistent evidence to support routine prophylactic antibiotics in elective anorectal surgery. Given the low baseline rates of SSIs and the lack of clear benefit, routine antibiotics are unlikely to be necessary in this context. However, selective antibiotic use remains appropriate for higher risk patients and may be appropriate in certain procedures, such as perianal abscess drainage. Caution is needed in interpreting this study's findings due to the small number of high‐quality studies, and further large‐scale randomized trials are needed to provide definitive guidance. Until then, a selective, risk‐based approach to antibiotic prophylaxis is recommended.

## Author Contributions

**Andrew Hill:** conceptualization, investigation, validation, methodology, writing – review and editing, supervision. **Maggie Wang:** investigation, writing – original draft, methodology, validation, visualization, writing – review and editing, formal analysis, project administration, data curation. **James Jin:** investigation, writing – original draft, methodology, validation, visualization, writing – review and editing, formal analysis, project administration, data curation. **Velia Men:** investigation, writing – original draft, methodology, validation, visualization, writing – review and editing, formal analysis, project administration, data curation.

## Funding

The authors have nothing to report.

## Ethics Statement

Ethical approval was waived due to the observational nature of this study.

## Conflicts of Interest

The authors declare no conflicts of interest.

## Supporting information


Supporting Information S1

## Data Availability

The data that support the findings of this study are openly available in Mendeley Data at https://doi.org/10.17632/7wyzsxkpnp.1.
