# Supplementary material for: Evidence Suggests Prophylactic Antibiotics May Be Unnecessary in Anorectal Surgery—A Systematic Review and Meta‐Analysis
Source: World J Surg. 2026 Feb 5;50(3):587–97. doi: 10.1002/wjs.70255 (PMC13006770; doi:10.1002/wjs.70255)
Supplement: Supplementary file 1 — Supporting Information S1 [file WJS-50-587-s001.docx]

| **Step** | **Search Terms** |
| --- | --- |
| 1 | “antibiotic” OR Anti-Bacterial Agents [MeSH] |
| 2 | “prophylaxis” OR Antibiotic Prophylaxis [MeSH] OR Pre-Exposure Prophylaxis [MeSH] OR Post-Exposure Prophylaxis [MeSH] |
| 3 | Anus Diseases [MeSH] OR Rectal Diseases [MeSH] OR Anal Canal [MeSH] OR Rectum [MeSH] |
| 4 | Hemorrhoidectomy [MeSH] OR Hemorrhoids [MeSH] |
| 5 | “anal fistula” OR Rectal Fistula [MeSH] OR “fistulectomy” OR “fistulotomy” OR “LIFT procedure” |
| 6 | “anal fissure” OR Fissure in Ano [MeSH] OR Sphincterotomy [MeSH] OR Lateral Internal Sphincterotomy [MeSH] |
| 7 | 3 OR 4 OR 5 OR 6 |
| 8 | 1 OR 2 |
| 9 | 7 AND 8 |

**Figure S1.** Search Strategy Used for the Systematic Review.

**Figure S2.** Forest plot showing the effect of prophylactic antibiotics on surgical site infection rates.

**Figure S3.** Forest plot showing the effect of prophylactic antibiotics on systemic infection rates.

**Figure S4.** Forest plot showing the effect of prophylactic antibiotics on postoperative bleeding rates.

**Figure S5.** Funnel plot assessing publication bias in the randomised controlled trials.

**Figure S6.** Funnel plot assessing publication bias in the observational studies.

**Table S1: MOOSE Checklist for Meta-analyses of Observational Studies**

| **Item No** | **Recommendation** | **Reported on Page No** |
| --- | --- | --- |
| Reporting of background should include | | |
| 1 | Problem definition | 2 |
| 2 | Hypothesis statement | - |
| 3 | Description of study outcome(s) | Abstract, 3 |
| 4 | Type of exposure or intervention used | 2-4 |
| 5 | Type of study designs used | 3 |
| 6 | Study population | 3-4 |
| Reporting of search strategy should include | | |
| 7 | Qualifications of searchers (e.g., librarians and investigators) | 1 |
| 8 | Search strategy, including time period included in the synthesis and key words | Abstract, 3 |
| 9 | Effort to include all available studies, including contact with authors | 4 |
| 10 | Databases and registries searched | 3, Fig 1 |
| 11 | Search software used, name and version, including special features used (e.g., explosion) | 4 |
| 12 | Use of hand searching (e.g., reference lists of obtained articles) | 4 |
| 13 | List of citations located and those excluded, including justification | 4, Fig 1 |
| 14 | Method of addressing articles published in languages other than English | 3 |
| 15 | Method of handling abstracts and unpublished studies | 4 |
| 16 | Description of any contact with authors | - |
| Reporting of methods should include | | |
| 17 | Description of relevance or appropriateness of studies assembled for assessing the hypothesis to be tested | 4-5 |
| 18 | Rationale for the selection and coding of data (e.g., sound clinical principles or convenience) | 4 |
| 19 | Documentation of how data were classified and coded (e.g., multiple raters, blinding and interrater reliability) | 4 |
| 20 | Assessment of confounding (e.g., comparability of cases and controls in studies where appropriate) | - |
| 21 | Assessment of study quality, including blinding of quality assessors, stratification or regression on possible predictors of study results | 4-5 |
| 22 | Assessment of heterogeneity | 5 |
| 23 | Description of statistical methods (e.g., complete description of fixed or random effects models, justification of whether the chosen models account for predictors of study results, dose-response models, or cumulative meta-analysis) in sufficient detail to be replicated | 4-5 |
| 24 | Provision of appropriate tables and graphics | Fig 1 |
| Reporting of results should include | | |
| 25 | Graphic summarizing individual study estimates and overall estimate | Fig 2, Tab 3 |
| 26 | Table giving descriptive information for each study included | Tab 1 |
| 27 | Results of sensitivity testing (e.g., subgroup analysis) | 6 |
| 28 | Indication of statistical uncertainty of findings | 10 |

| **Item No** | **Recommendation** | **Reported on Page No** |
| --- | --- | --- |
| Reporting of discussion should include | | |
| 29 | Quantitative assessment of bias (e.g., publication bias) | 8, Fig S4-5 |
| 30 | Justification for exclusion (e.g., exclusion of non-English language citations) | - |
| 31 | Assessment of quality of included studies | 8, 10 |
| Reporting of conclusions should include | | |
| 32 | Consideration of alternative explanations for observed results | 10 |
| 33 | Generalization of the conclusions (i.e., appropriate for the data presented and within the domain of the literature review) | 9-10 |
| 34 | Guidelines for future research | 9, 11 |
| 35 | Disclosure of funding source | 1, 11 |

*From*: Stroup DF, Berlin JA, Morton SC, et al, for the Meta-analysis Of Observational Studies in Epidemiology (MOOSE) Group. Meta-analysis of Observational Studies in Epidemiology. A Proposal for Reporting. *JAMA*. 2000;283(15):2008-2012. doi: 10.1001/jama.283.15.2008.
